# Rheumatic Nodules

**Published:** 1908-11-21

**Authors:** 


### Diseases of Children.

### RHEUMATIC NODULES.

Many points of interest arise in the study of the so-called rheumatic nodules and their association with rheumatism, chorea, and severe heart affections, their age-incidence, their pathology, and their diagnostic and prognostic import. They are described as subcutaneous fibrous nodules, composed of collections of inflammatory lymph among fibrous tissue, analogous to the fibrinous deposits in endocarditis and pericarditis. In shape they are round or oval. In size they vary from a pin's head to a pea, but occasionally they reach the size of a large marble. In number they vary considerably, being scanty or very numerous. When small they are much better felt than seen. They are neither painful nor tender. They are commonly attached to the fascia, tendon sheaths, or underlying fibrous tissue about the bony prominences and joints, especially the margins of the patella, olecranon, malleolus, and finger joints; on the extensor tendons of the hands, fingers, and toes; over the spinous processes of the vertebræ and scapulæ. Sometimes on the scalp they are extraordinarily large and numerous. Small ones about the joints are most visible when the joint is flexed. They are usually transitory, coming and going quickly; but they may come out in crops of a few weeks' duration, or even in successive crops in the same situations for several months. They are not adherent to the skin, never suppurate, and eventually disappear and leave no ill-effects behind.

These nodules were first described by Barlow and Warner in 1881 as a sign of rheumatism. Subsequently Garrod pointed out that they are apt to be associated with severe types of heart lesion. In more recent years it has been found that they occur even independently of cardiac mischief. Hadden recorded such a case in 1889, rheumatic fever and endocarditis developing subsequently. Thursfield in 1901 found them in a boy, aged nine years, with rheumatic periostitis, slight rheumatic symptoms, and a dilated heart. Carpenter (Reps. Soc. Dis. in Children, 1901, i., 26) showed an infant aged seventeen months, whose hands had been covered with multiple fibrous nodules of this type for three months. For two years subsequently fresh nodules appeared, some disappeared, and throughout there was no evidence and no symptom suggestive of rheumatism. Lees in 1902 showed a boy, aged nine years, with nodules and no heart lesion, the nodules disappearing after three months' treatment with salicylates. Attention must also be drawn to their occurrence in osteo-arthritis, a disease in which they are usually larger and more persistent, though the matter of size is of no value from an ætiological point of view. The writer can recall to mind their occurrence in large numbers in a girl with general tuberculosis. In chorea we are met with the remarkable fact that nodules are not present unless there is associated rheumatism. This is very curious, if we accept the view that nodules are an important indication of rheumatism in early life, and that chorea is a manifestation of rheumatism. Again, in relation to age it is somewhat surprising that both nodules and chorea are common in childhood, rare when youth is reached, and very uncommon after the age of twenty-one years. One would expect, therefore, that nodules and chorea would be frequently associated if they are both rheumatic manifestations. The rarity of nodules in adults is striking, for rheumatism increases in frequency and is most common from ten to twenty years of age, while nearly fifty per cent. of the cases are between twenty and forty years old. Poynton and Paine found the organism to which they ascribe rheumatic fever in these nodules. Assuming this to be the true infective organism it is strange that multiple rheumatic nodules can occur without other evidence of rheumatism. Either the organism is not the causative agent or these nodules can arise from other causes. The diplococcus rheumaticus was warmly welcomed when it was described by Wassermann, and subsequently by Poynton and Paine; but since then so much doubt has been cast on its specific characters that we should be rash to assume it as the cause of rheumatic nodules.

From the point of view of diagnosis the rheumatic nodule becomes of some value because of its frequent association with rheumatism in early life. If such nodules are found, even in the absence of all other history, signs, or symptoms of rheumatism, anti-rheumatic treatment should be adopted and very careful watch directed to the heart, for, as is well known, rheumatic heart affections are constantly very insidious in their onset in children. On the other side we must remember that the evidence so far available does not justify us in stating that where there is a nodule there is rheumatism. In a way these nodules suggest the possibility of some relations between true rheumatism and osteo-arthritis. A girl of eleven years under the writer's care has well-marked nodules, chorea, morbus cordis, and a joint affection suggestive of osteo-arthritis rather than true rheumatic fever. In adults, too, it is not uncommon to find osteo-arthritis associated with a chronic form of pericarditis or definite endocarditis.

From a prognostic standpoint these nodules are relatively unimportant or of grave consequence according as the physician attaches gravity to the statement that nodules are apt to be associated with serious forms of heart disease. That this is true in some cases may be admitted. In the majority it will be found that they are of no prognostic value, for they often occur without cardiac mischief, and sometimes with no evidence of rheumatism. The immediate prognosis, therefore, depends on the severity of the disease with which they are associated. The ultimate prognosis must be guarded as long as we accept the rheumatic theory of their causation, for such children must be treated as liable to subsequent attacks of rheumatism and its complications.

No special treatment need be adopted in the ordinary way for the nodules themselves. In those lasting for months it is worth while trying the effects of general and local massage, applied gently, hydrotherapeutics, hot air baths, and electrical treatment by various rays or ionisation.

